# Open-source, low-cost 3D-printable testbed for in-body optical wireless communications research

**DOI:** 10.1016/j.ohx.2026.e00744

**Published:** 2026-01-20

**Authors:** Syifaul Fuada, Lukasz Surazynski, Mariella Särestöniemi, Teemu Myllylä, Marcos Katz

**Affiliations:** a Centre for Wireless Communications, Faculty of Information Technology and Electrical Engineering, University of Oulu 90570 Oulu, Finland; b Infotech Oulu 90014 Oulu, Finland; c Research Unit of Health Sciences and Technology, Faculty of Medicine, University of Oulu 90220 Oulu, Finland; d Optoelectronics and Measurement Technique Research Unit, Faculty of Information Technology and Electrical Engineering, University of Oulu 90570 Oulu, Finland; e Medical Research Center (MRC) Oulu, University of Oulu 90014 Oulu, Finland

**Keywords:** In-body devices, In-body communication, Optical wireless communication (OWC), 3D-printable testbed

## Abstract

This hardware paper introduces an experimental testbed for in-body optical wireless communication (OWC) studies. The conventional version often relies on bulky optical benches and costly supporting equipment, which are often cost-prohibitive for many research institutions. The proposed testbed featured a small footprint, lightweight, a vertically aligned optical path (with fixed optical component placement), and ambient light shielding. It can be printed using commercial 3D printing, reducing costs compared to conventional optical benches. The 3D-printable testbed consists of a box-like chassis that securely positions a near-infrared (NIR) LED TX at the top and a photodetector RX at the bottom, with a tissue sample (*e.g.*, ex-vivo porcine tissue or a tissue-mimicking phantom) held firmly in between. All design files, including CAD and STL formats, along with detailed assembly instructions, are made openly available. The inherent design structure enables faster alignment, and the shields can effectively protect against exposure to indoor ambient light (*e.g.*, typical laboratory lighting), thereby improving experimental reliability. The modular nature of the testbed allows for easy customization to accommodate sensors of different wavelengths and different tissue models. The proposed testbed offers practical benefits and an accessible solution for researchers conducting in-body OWC studies, especially when access to high-end optical equipment is limited.

## Graphical abstract

**Figure:**
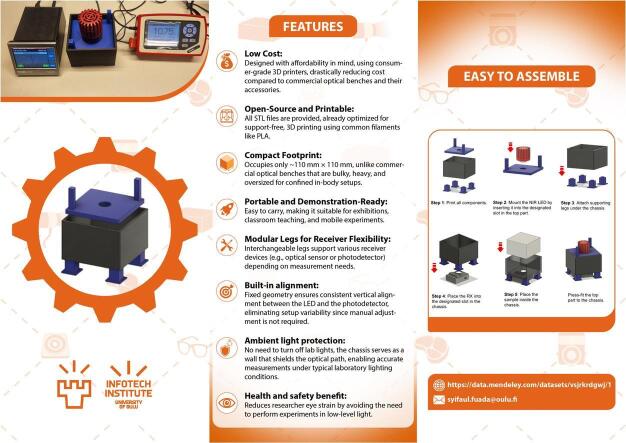

**Specifications table:** 

Hardware name	*Open-source, Low-cost 3D-Printable Testbed for Optical-based In-body Communication Research*
Subject area	• *Open-source alternatives to Existing Experimental Test Equipment and Infrastructure.* • *Biomedical Engineering.* • *Optical Wireless Communication (OWC).* • *Photonics.* • *Educational Tools for Baseline Measurement.*
Hardware type	• *3D-Printed Experimental Testbed.* • *Measurement Fixture*.
Closest commercial analog	*The closest commercial platforms to the proposed testbed include standard optical laboratory components, i.e., optical breadboards/benches (e.g., MB3060/M, Thorlabs, USA) + post holders (e.g., MSL2 or MSL3, Thorlabs, USA) + lens tubes (e.g., SM1TC, Thorlabs, USA).*
Open source license	*This work is licensed under the Creative Commons Attribution-ShareAlike 4.0 International License. To view* *a copy of this license, visit**http://creativecommons.org/licenses/by-sa/4.0/*
Cost of hardware	*The cost of the materials used in the 3D-printable testbed is approximately $6.6.*
Source file repository	*Repository: Mendeley* *URL:**https://data.mendeley.com/datasets/vsjrkrdgwj/1* *https://doi.org/10.17632/vsjrkrdgwj.1*

## 1 Hardware in context

### 1.1 In-Body Optical Wireless Communication (OWC) Research

Statistics reported in [1] indicate that tens of millions of people experience different physical diseases annually, including chronic and congenital conditions that serve as ticking time bombs, presenting serious risks to patients and their families. In recent decades, advances in electronics and manufacturing have driven significant progress in modern medicine. This progress has led to innovative diagnostic and monitoring tools, including infrared light–based therapies and in-body electronic devices (IEDs). Based on the analysis and industry forecast report of the global medical implants market, a wide variety of products are available. Among biomedical telemetry devices, IEDs have attracted significant attention for their ability to acquire and store physiological data in real time [2].

IEDs are a type of medical device that can be broadly categorized into three groups: implantable, ingestible, and injectable medical devices [3]. As demand for these systems increases, their features have expanded, particularly by incorporating wireless communication capabilities. These wireless links enable bidirectional communication: from the IED to the external world (in-body-to-on-body link) and from the external world to the IED (on-body-to-in-body link). Communication with IEDs, whether transmitting data from inside the body (backward telemetry) or receiving commands from external sources (forward telemetry), is known as in-body communication. Applications for backward telemetry include real-time monitoring of organ health and other related medical data collections. Meanwhile, the forward telemetry application includes invasive adjustments to IED operations based on the patient's condition, administering doses or therapies, and updating software, among other functions. More efficient and ultra-reliable wireless communication solutions are critically needed for in-body communication to meet stringent performance requirements. Traditional radio frequency (RF) techniques have been extensively studied for in-body communication. Particularly, RF communication benefits from well-established standards. For instance, the Medical Implant Communication System (MICS) band (401–406 MHz, core 402–405 MHz) defines a low-power, short-range, relatively high-data-rate link widely accepted for transmitting diagnostic and therapeutic data to and from IEDs. This frequency band has been extensively studied to support mobile, reliable, and comfortable communication systems that improve human life [4]. However, RF communication now operates in an increasingly congested spectrum, where overlapping services and dense device activity can cause signal interference and reduced link reliability, possibly leading to data loss [5]. This problem is especially crucial in hospital environments, where numerous medical devices may operate simultaneously and share the same RF bands [6]. It is essential to note that many everyday devices, not just medical equipment (*e.g.*, mobile phones, security scanners, and other wireless devices), also emit RF waves [7]. In hospital environments, such devices could disrupt radio-sensitive medical equipment. Isolating IEDs from these RF waves is challenging, further motivating the exploration of non-RF communication modalities for in-body applications.

**Fig. 1:**
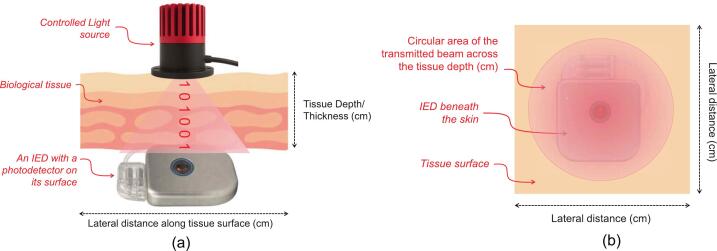
Illustration of optical characteristics for an in-body OWC system: (a) Side view of the biological tissue. The light source penetrates only a few millimeters to centimetres into tissue; (b) Top view of the tissue surface. The circular area shows the illuminated region of the NIR LED. An optical TX beam typically illuminates only a limited spot on the surface, and its emission remains confined to the desired IED area rather than radiating broadly into surrounding tissue, as is typical for RF transmissions. These unique advantages make OWC more advantageous in hospital settings, such as reducing the risk of eavesdropping on IEDs. The x–y plane represents the lateral tissue dimensions, demonstrating that a small tissue sample is sufficient for the in-body OWC experiment. Conceptual design (base layout) by the authors; graphical assets generated with an AI tool (ChatGPT/DALL·E, *OpenAI*, USA) and subsequently edited by the authors.

Optical wireless communication (OWC) has recently emerged as a promising alternative for such applications due to its favorable characteristics [8], including high bandwidth, license-free operation by leveraging the underused optical spectrum, immunity to electromagnetic interference (EMI), potential for miniaturization, and low power consumption. On the other hand, OWC offers a private and secure method for data exchange across biological tissues [6]. The rationale is that the in-body optical links are inherently short-range and focused, as illustrated in Fig. 1(a) and (b), respectively. Near-infrared (NIR) light, in particular, offers favorable characteristics for in-body OWC applications due to its safe tissue profile [7], [9]. Importantly, NIR light in the 650–1100 nm range, often referred to as the “optical window,” “therapeutic window,” or “NIR window,” offers the greatest penetration depth in biological tissue. Within this band, absorption by major chromophores such as water and hemoglobin is relatively low, enabling photons to penetrate several millimeters to a few centimeters into soft tissue. These characteristics make the NIR window particularly suitable for minimally or noninvasive solutions in most biological systems [10].

For optical transmitter (TX) in in-body OWC systems, Light-emitting diodes (LEDs) are a favorable choice due to their larger spot or beam profiles compared to those of laser sources, which are expected to improve tolerance to misalignments and pointing errors. LEDs are also considerably more cost-effective than laser sources [11]. Additionally, LEDs are generally safer for biological tissues, pose a lower photothermal risk, and are widely used in safe therapeutic applications, such as Photobiomodulation (PBM) [9], further supporting their clinical relevance.

Many previous studies have assessed the feasibility of in-body OWC systems using *ex-vivo* experimental models, such as [12], [13], [14], [15]. These often include biological tissues, such as porcine samples (*e.g.*, skin, fat, muscle), which replicate light transmission through human tissues [16], [17]. Ex-vivo setups offer a controlled and ethical alternative to in-vivo testing, which typically involves live animal models and raises concerns about animal welfare. Despite not involving living subjects, *ex-vivo* models retain the key optical properties of actual biological tissues, making them essential for validation before clinical implementation. Beyond wavelength-dependent absorption and scattering, both tissue thickness [18] and alignment precision [19] also strongly affect in-body optical link performances. Consequently, the alignment configuration is particularly critical for establishing baseline measurements in the context of in-body OWC systems.

### 1.2 Existing Test Bed: Technical Challenges

A conventional optical bench setup is commonly used in OWC research, including studies focused on in-body communication applications. Optical benches are also a common baseline device for experiments such as spectroscopy and optical imaging. In standard optical laboratory setups, an optical bench is employed to mount components such as lenses, emitters, and detectors. The hole-pattern interface allows these elements to be manually repositioned, supporting modular experimental configurations while preserving stable mounting along an optical axis. Optical benches, on the other hand, are typically constructed from rigid metallic materials, such as aluminum or steel, to ensure long-term stability and suppress vibration. Due to these requirements, optical benches are generally heavy and bulky.

**Fig. 2:**
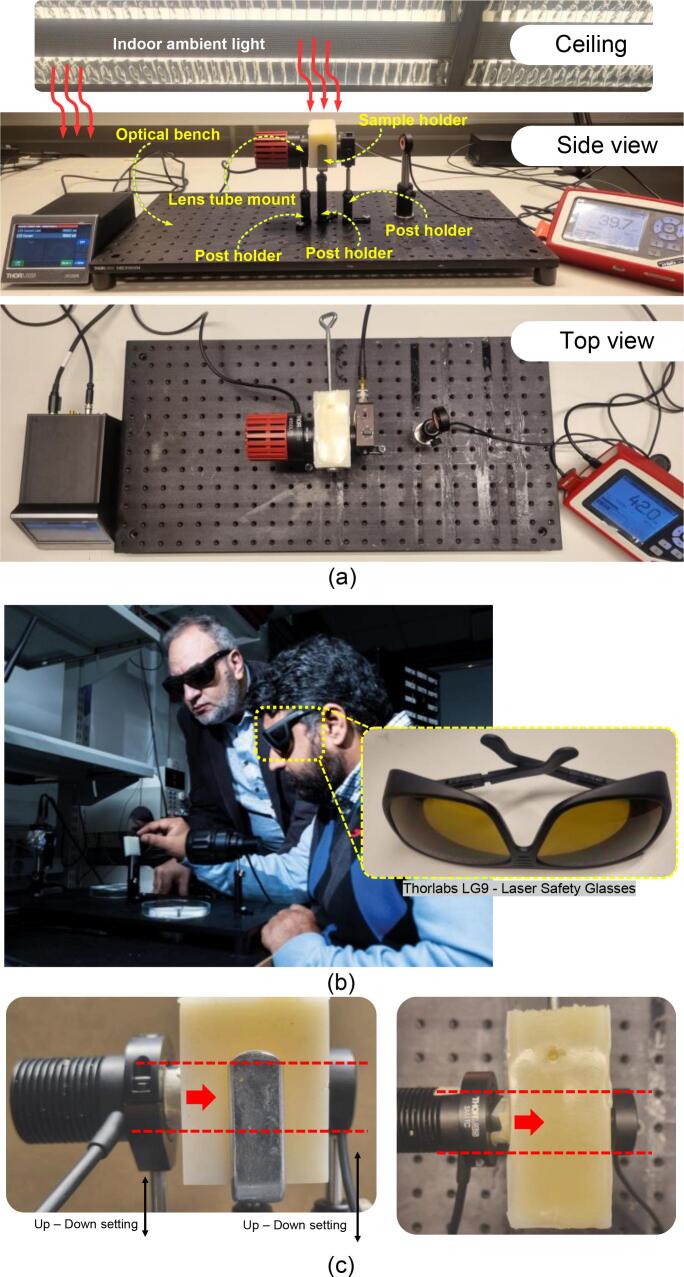
(a) Former experimental testbed, demonstrating the use of an optical bench with lens tubes and post holders. The optical bench is a stainless-steel optical baseline used for general photonics experiments and serves as a mounting platform for optical components. While highly stable, it is typically heavy, expensive, and not optimized for confined or vertically aligned NIR transmission through a biological tissue sample. It measures more than 300 mm × 600 mm, which is oversized for in-body OWC experiments and far exceeds the spatial requirements for small tissue samples. Additionally, it is also not easily transportable. Meanwhile, the post holders require careful manual alignment and lack ambient light shielding. The lens tubes, on the other hand, while they do provide a reliable holder for mounted LEDs integrated with post holders, require an additional cost. This makes them less accessible for institutions with limited funding. (b) The illustration of the experimental work conducted in our laboratory followed the safety protocol; a photograph was obtained from [35]. Prof. Marcos Katz and his researcher are shown aligning an optical phantom while wearing certified laser safety glasses (LG9, Thorlabs, USA), which protect against NIR emissions. (c) Practical challenges in the previous optical setup showed manual alignment between the TX and RX. The side view (left): dashed lines illustrate the required strict alignment between the TX’s central beam and the RX’s active area through the optical phantom. Top view (right), emphasizing precise physical centering of the TX relative to the RX.

Fig. 2(a) illustrates the typical experimental setup used in our laboratory for in-body OWC research, which was operated in a standard optical laboratory environment and fully exposed to indoor laboratory lighting (which serves as the ambient light source). The testbed consists of an NIR LED as the TX source, a sample (*i.e.*, ex-vivo porcine sample or tissue-mimicking phantom) placed in a holder, and a photodetector as the receiver end (RX) positioned on the opposite side. The TX, RX, and sample holder were placed on an optical bench. Calibrated optical instrumentation from Thorlabs was used. Thorlabs offers a wide range of standardized optoelectronic components that are well-suited for early-stage research and prototyping. For instance, their catalog includes mounted LEDs across multiple wavelength bands—including NIR, visible, and ultraviolet—which allows researchers to easily select and interchange sources based on experimental requirements. They are also designed to be physically and electrically compatible with dedicated Thorlabs’ LED drivers, forming a unified ecosystem that streamlines experimentation and enables systematic exploration of multiple optical parameters across diverse scenarios.

Fig. 2(b) illustrates the research protocol used in the experiments, including the use of protective eyewear to safeguard researchers from NIR radiation. Because NIR light is invisible to the human eye, accidental exposure may go unnoticed, highlighting the importance of proper eye protection throughout all measurement procedures.

We encountered several practical challenges when using the conventional testbed (Fig. 2a).

First, establishing a strict alignment between the TX and RX can be time-consuming, as it requires careful manual positioning and mechanical assembly on an optical bench using post holders, screws, and a screwdriver. As shown in Fig. 2(c), the RX had to be precisely collinear with the TX—when viewed from above, all components needed to be centered along the same axis to maintain proper TX–RX alignment.

Although the adjustable holders offer mechanical flexibility for positioning the TX and RX, they also make it more difficult to achieve precise, repeatable alignment across multiple assemblies. Even small lateral or angular offsets between the TX and RX can contribute to degrading the received optical power slightly [19].

Second, experiments often need to be conducted under low-intensity conditions to minimize interference from indoor ambient lighting. When the laboratory room lights are on and the phantom is uncovered, it absorbs a small portion of the ambient light, slightly increasing the background illumination at the receiver. This, in turn, introduces additional optical noise into the measurements (see Appendix, Fig. 17). Operating in a fully darkened environment also introduces safety and ergonomic concerns, particularly during long experimental sessions, as prolonged work in darkness can contribute to eye strain, visual discomfort, and reduced productivity among researchers. Prolonged work in low-light conditions increases visual strain and may cause headaches, blurred vision, and eye fatigue [20]. Furthermore, conducting experiments in a fully dark environment while wearing protective glasses, as illustrated in Fig. 2(b), not only causes discomfort but also creates ergonomically challenging conditions for the researchers.

### 1.3 Targeted Research Problems

This hardware paper addresses the following targeted research questions:

*RQ1*: How can a testbed be designed on a small footprint with adequate indoor ambient-light shielding to enable reliable optical measurements under standard laboratory lighting, without the need for a darkroom?

*RQ2*: How can the compact testbed be designed to be 3D-printable and easy to assemble (*i.e.*, modular) using only low-cost materials and widely available desktop 3D printers?

*RQ3*: How well does the testbed design support strictly consistent TX–RX alignment and reproducible measurement results across multiple sessions involving repeated unplugging, re-plugging, and reassembly?

### 1.4 Review of the Similar Self-Design Testbed

Over the past decade, researchers have investigated in-body communication using a wide range of experimental testbeds, each designed and optimized for the specific communication modality under investigation, *e.g.*, ultrasound, capacitive, inductive, microwave, optical, and other approaches. To the best of our knowledge, there is currently no established standard or widely accepted reference architecture for testbed design in in-body communication research, given the diverse testbed designs reported in the literature. Rather, most designs are custom-made, specifically tailored to the experiment's context.

To contextualize our testbed design, we compared it with several representative in-body communication systems from the literature, focusing on microwave and optical domains. Table 1 summarizes the selected prior testbed examples by presenting both photographs and schematics. For works that did not report a schematic, we reconstructed 2D layouts from the original images to provide clearer visualization.

**Table 1:** Comparison of existing in-body communication testbeds.

**Label**	**Image Source**	**Original photographs**	**Testbed architecture**
Example *#1*	[36]		
Example *#2*	[37]		
Example *#3*	[38]		
Example *#4*	[39]		
Example *#5*	[40]		
Example *#6*	[13]		
Example *#7*	[41]		
Example *#8*	[42]		
Example *#9*	[6]		
Example *#10*	[15]		
Example *#11*	[12]		
Example *#12*	[43]		
Example *#13*	[44]		
Example *#14*	[45]		

Examples *#1–5* present testbeds in the microwave domain. The testbed emphasizes the use of coaxial probes, embedded antennas, and *ex-vivo* porcine sample tissues, positioned in open molds or surrounded by absorptive material fixtures. These testbeds often employ horizontal arrangements and are typically optimized for electromagnetic propagation studies. Examples *#6–14* present a selection of recent testbeds for in-body OWC studies. These platforms usually rely on either conventional horizontal optical bench arrangements or compact, custom-built setups with a minimum footprint.

The side-by-side comparison in Table 1 highlights differences in geometry and intended purpose, providing a clear visual contrast between prior testbed designs and those proposed in this work. As shown in Examples *#6–14*, prior optical testbeds tend to use open-frame designs and provide only limited shielding against ambient light and environmental interference. It should be noted that the 2D reconstructions presented in this hardware paper (Examples #2, #5-#6, #8-#14) are qualitative schematics illustrated by the authors based on visual inspection of the referenced photograph and are not exact reproductions of the original designs. They are meant only to demonstrate the general geometry and layout of TX – RX for comparison.

The testbed designed in this study primarily addresses challenges related to a compact footprint, lightweight design, alignment, and protection against ambient light. We expect that such testbed capabilities not only reduce alignment setup time but also maintain user comfort and eye health by eliminating the need to turn OFF laboratory lighting.

To design built-in vertical alignment, the TX is mounted at the top and pressed against the phantom. Meanwhile, the RX is fixed at the bottom, ensuring consistent vertical alignment and minimizing optical power loss due to misalignment. To design the testbed with a light-shielding enclosure, the sample and optical path are isolated from ambient light by the cubic geometry box. These features allow researchers to work safely and comfortably with the laboratory room lights on without compromising measurement reliability. The proposed testbed offers a much smaller footprint while retaining the beneficial features mentioned earlier.

Importantly, the testbed also offers a cost-effective solution that implements 3D principles while ensuring experimental quality, thereby practically minimizing experimental costs for in-body OWC experiments. Advances in 3D printing are well-known for enabling the design and construction of custom scientific instruments [21], that are both cost-effective and accessible with consumer-grade equipment.

This hardware paper aims to provide scientific documentation of the design and its practical use, with the expectation of contributing to the growing need for experimental testbeds in the emerging field of in-body OWC studies. Furthermore, we expect that this proposed design can be easily replicated and adapted by other researchers, as the testbed is designed simply for easy reproduction.

## 2 Hardware description

### 2.1 Existing Testbed: Cost-related Issues

This section elaborates on the limitations of our previous testbed (Fig. 2a) from a cost-constraint perspective, highlighting the need for a lower-cost alternative. As mentioned above, previously we employed a solid aluminum optical breadboard (MB3060/M, *Thorlabs*, USA) measuring 300 mm × 600 mm × 12.7 mm for the base. It is priced at approximately €294.30 (equivalent to $337) excluding VAT; details are presented in Table 2. Furthermore, it is bulky and relatively heavy (6.82 kg) and requires additional mounts and fixtures for each component (LEDs, photodiodes, holders, etc.). The price was calculated based on the currency exchange rate in WISE (*https://wise.com/gb/currency-converter/eur-to-usd-rate?amount=294*) as of June 23, 2025.

**Table 2:** Specification of the current testbed: Optical bench.

**Component name**	**Model**	**Cost**	**Weight**	**URL**
Optical Bench	MB3060/M − Aluminum Breadboard, 300 mm x 600 mm x 12.7 mm, M6 Taps	€ 294,30	6.82 kg	*https://www.thorlabs.com/thorproduct.cfm?partnumber=MB3060/M*

We also have three post holders for TX, RX, and samples (MSL3 and MSL2, Thorlabs, USA), as well as a lens tube mount (SM1TC, Thorlabs, USA) to hold the mounted LED; details are presented in Table 3.

**Table 3:** Specification of the current testbed: Accessories.

**Component name**	**Model**	**Cost**	**Weight**	**URL**
Post holder	MSL3 − Swivel-Base Mini-Series Post Holder, 3″ (76 mm) Tall, 1/4″ (M6) Slot	€ 46.80	0.01 kg	*https://www.thorlabs.com/thorproduct.cfm?partnumber=MSL3*
Post holder	MSL2 − Swivel-Base Mini-Series Post Holder, 2″ (51 mm) Tall, 1/4″ (M6) Slot	€ 41.88	0.03 kg	*https://www.thorlabs.com/thorproduct.cfm?partnumber=MSL2*
Lens tube mounts	SM1TC − Clamp for SM1 Lens Tubes and C-Mount Extension Tubes	€ 48,80	0.02 kg	*https://www.thorlabs.com/thorproduct.cfm?partnumber=SM1TC*

The SM1TC lens tube clamp and mounting post holders cost approximately € 48.80 and € 41–47, respectively.

Using a full-scale optical bench (for example, our previous optical bench setup occupied a footprint of over 300 mm × 600 mm) proved to be oversized and excessive for in-body OWC research [22]. In such applications, the effective transmission distance of NIR light through biological tissue is typically limited to a few millimeters to centimeters, as illustrated in Fig. 1(b).

In particular, a large benchtop system for an alternative testbed is unnecessary. Instead, a small-footprint design is considerably more efficient, aiming to reduce overall bulk and weight while lowering fabrication costs. Importantly, the alternative testbed should also eliminate the need for commercial holders (*i.e.*, mounting hardware) by integrating a dedicated optical component into the 3D-printed structure. By providing a fixed, vertically aligned configuration that ensures consistent optical coupling between the TX and RX, the design not only minimizes misalignment but also improves measurement consistency and offers a low-cost alternative to conventional optical bench assemblies. Moreover, by sharing all design files in an open and 3D-printable format, the platform could be easily reproduced and adapted by other researchers.

### 2.2 Open-source Optical Testbed

The development of 3D-printable, cost-effective, and open-source testbeds as alternatives to commercial testbeds is not a particularly new approach. In the context of optical research, such testbeds help significantly reduce instrumentation costs, while in educational settings, they offer benefits for teaching and hands-on learning. Open-source principles make experimental tools more accessible at low cost. For example, [23] demonstrated the feasibility of producing a 3D-printable open-source optics lab for teaching and research, enabling the low-cost assembly of optical benches, lens holders, and other photonics hardware using standard plastic filament and consumer-grade 3D printers, thereby substituting for commercial products. Their designs were primarily suited for optical rail systems, which are widely used in physics education but less applicable to chemistry applications. Building on this, [24] focused on expanding the 3D-printable optical hardware ecosystem for chemical education, introducing a set of modular optical mounts, posts, and holders designed to interface with standard optical breadboards. Their designs enabled undergraduate students to assemble a visible spectrophotometer using visible LEDs and photodiodes, and provided teachers with benefits to teach optical absorption and fluorescence principles. Both references [23], [24] illustrate the potential of 3D printing in optics for educational purposes.

Later, in [25] developed a 3D-printed opto-mechanical cage system with enclosure, as a low-cost alternative to commercial optical alignment setups, which is advantageous for Raman spectroscopy and Interferometry experiments. Traditional hardware of this kind is often built from precision-machined metal and can be prohibitively expensive for teaching labs. The hardware featured 3D-printed rods and lens holders, intended for general-purpose beam alignment in over-air optical experiments, and had a large footprint. Later, in [26] developed a 3D-printed motorized rotation stage system that provides precise angular control of optical elements (*e.g.*, mirrors or polarizers) in experiments, fully controlled by a microcontroller. This hardware uses an open-source approach, enabling fabrication with common 3D printers and readily available electronics. Additionally, it is a low-cost alternative to the high expense of commercial motorized stages used in beam steering, optical alignment, and polarization control.

Meanwhile, our 3D printable testbed is specifically optimized for in-body OWC research.

### 2.3 Advantages of the Proposed 3D-Printable Testbed

The proposed 3D-printable testbed for in-body OWC research embodies the following properties:

• **Openness**: The design supports open hardware principles with STL files freely available in the repository, enabling full reproducibility, modification, and reuse by the research community.
• **Cost-effectiveness Solution**: A typical optical bench with standard optomechanical components (*e.g.*, post holders and lens-tube mounts) usually costs much more than €100, as summarized in Table 2, Table 3. In comparison, the proposed 3D-printable testbed can be assembled entirely from affordable consumer materials and readily available parts. The complete setup—including the top part, chassis, and detachable legs—can be printed for a total cost well under €100, depending on the printing method. This represents over 90 % cost savings compared to commercial optical benches, while still providing the necessary dedicated features for in-body OWC experiments, *i.e.*, alignment precision and ambient-light shielding. Moreover, the design eliminates the need for expensive mounting accessories, such as the SM1TC lens-tube clamp, to secure the LED assembly. Note that measurement tools such as the optical power meter and optical sensor are shared between both setups and are not included in the platform's cost.
• **Portability**: Unlike bulky optical benches, our 3D-printed testbed has high portability. It can be easily transported to different locations. The lightweight design makes the testbed easily portable, which is highly beneficial for conference exhibitions, academic demonstrations, and mobile experimentation scenarios where simplicity and space efficiency are critical. The proposed testbed occupies approximately 110 mm × 110 mm. This ∼ 90 % reduction in dimension compared to the optical bench that we already have makes the setup better in reflecting the spatial constraints of realistic in-body environments.
• **Ease of use**: The fixed-align design eliminates the need for manual adjustments of optical mounts, unlike traditional optical benches that often require careful and time-consuming alignment settings, thereby significantly reducing setup time and improving experimental repeatability. The built-in mechanical guides in the 3D-printed testbed ensure consistent vertical alignment of the TX and RX components, just by pressing the TX directly against the top of the tissue sample and securing the RX at the bottom.
• **Ambient light protection**: Our compact cubic 3D-printed testbed allows for measurements under standard laboratory lighting conditions, thereby benefiting health and safety, as it reduces researchers' eye strain by avoiding the need to perform experiments in darkness.
• **Benefiting educators and students**: Our 3D-printed testbed is an Ideal solution for benchtop or educational use, unlike bulky aluminum optical tables with their accessories, as it is an affordable tool for classroom use (*e.g.*, teaching optical alignment and tissue optics), thereby promoting low-resource labs that are more accessible for students and educators.
• **Simple cubic geometry**: Our 3D-printable box (*i.e.*, cube enclosure) houses an experimental sample. Specifically, we chose the cube for its flexibility in shaping tissue samples, which ensures universal sample placement. As shown in many phantom models, the cube is a popular form for layered tissue-mimicking phantoms because it is easy to produce and can be divided into smaller and uniform sections. Cubes are simple to cut, stack, and align, which helps when building layered structures with specific thicknesses. Another benefit of a cube is its ability to balance the chassis properly with legs at each corner. Although cylindrical shapes might be advantageous for optical alignment and compatibility with standard optical components, they are less practical in this case, as reshaping tissue or phantom blocks into circles would be inefficient and wasteful, potentially compromising uniformity. Ensuring consistent tissue thickness and a uniform surface is vital to the experiment. The external shape does not affect optical performance because the internal design naturally aligns the NIR LED, the tissue sample, and the receiver. We anticipate that the cubic enclosure will provide the same optical accuracy as a cylindrical one. Additionally, although Thorlab’s parts are cylindrical, they fit well into our design.
• **Enabling future modelling or simulation works**: All components are available as STL files in the repository, which might allow not only physical replication but also potential reuse in numerical models. Our 3D-printable testbed is designed for use with biological tissue samples up to 10 × 10 × 7 cm, which corresponds to the typical size range of actual tissue sample dimensions or tissue-mimicking blocks used in optical experiments. Because the NIR optical beam for in-body OWC is highly localized and illuminates only a small area of the sample, such a compact tissue volume is fully sufficient for characterizing optical propagation, attenuation, and data or power transfer in this context. In principle, the STL geometries can be imported into Monte Carlo or diffusion-based light-transport simulators to model photon propagation through the testbed. As a concrete example, MATLAB can recreate the precise enclosure by importing the STL files into the Partial Differential Equation (PDE) Toolbox using the command: *importGeometry(“file.stl”)*, which generates an imported geometry object suitable for meshing and simulation. Once a well-defined tissue-block model is readily available and a suitable diffusion-based light-transport program is established in MATLAB, the imported geometry will serve as the computational domain for simulations. This may enable future work to correlate simulated photon paths and fluence distributions with experimentally measured received optical power using the same 3D-printable testbed geometry.

## 3 Design files summary

In general, our 3D-printable testbed consists of three parts that can be manufactured via filament printing from various materials: the top part, the body (chassis), and the leg part, as detailed in this section.

In this hardware paper, we propose polylactic acid (PLA) due to its biodegradability, ease of machining, rigidity, and accessibility. All proposed parts could be printed with 30 % infill and no additional supports, thereby lowering the cost of the testbed and reducing printing time. Furthermore, interchangeable supports (in this study, 3- and 20-mm legs) enable the main part (*i.e.*, chassis and top parts) to be lifted to the desired height for different optical sensors, while ensuring the main part remains on a flat surface and maintains a consistent experimental setup.

It should be noted that the 3D-printable testbed is currently designed to support only mounted LEDs, optical sensors, and photodetectors from the specific company. The mounted NIR LED involves mounted M810L3, MBB2L1, M850LP1 (*Thorlabs*, USA), and so on. In our laboratory, we have an optical sensor (S121C, *Thorlabs*, USA) and a photodetector (PDA36A-EC, *Thorlabs*, USA), both with the same ring diameter.

The 3 mm legs were used with thin and compact RX-type sensors, such as S120VC, S120C, S121C, and S122C (*Thorlabs*, USA), which require minimal clearance. In contrast, the 20 mm legs were employed for thicker RX sizes, such as the PDA36A-EC, providing adequate vertical space under the chassis part and maintaining proper alignment along the optical axis. For different RX thicknesses, for instance, PDA36A2 (*Thorlabs,* USA), it is not necessary to reprint the entire enclosure. Instead, it is recommended to modify and reprint only the support legs to the desired height. This modular approach simplifies adaptation to various sensor types while maintaining optical alignment and structural consistency. A new design for the leg modules will be provided in the repository, enabling easy customization of leg height to match specific detector thicknesses.

**Fig. 3:**
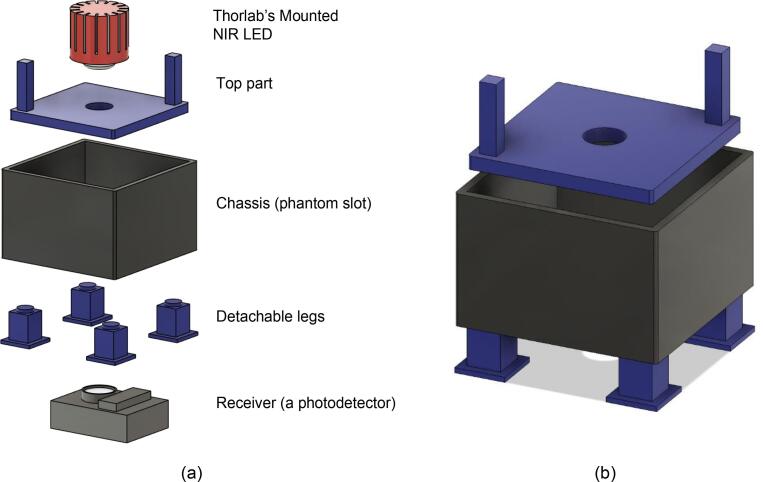
(a) Visual depiction of a cost-effective 3D-printed testbed designed for research in an in-body OWC experiment. The geometry of the design ensures vertical alignment between the TX, RX, and sample; (b) a “*complete testbed.f3d*” file.

The summary of the design files is provided in Table 4. All associated electronic files are available in the repository (*i.e.*, *https://data.mendeley.com/datasets/vsjrkrdgwj/1*), including computer-aided design (CAD) files ready for 3D printing. The required materials for printing are listed in the following section, and a detailed description of each component’s function is provided in Section 5 (Built instruction). Fig. 3(a) is the exploded view of the proposed 3D-printed testbed for the in-body OWC experiment. The design consists of:

**Table 4:** Design files summary.

**Design file name**	**File type**	**Open-source license**	**Location of the file**
*Top*	*.stl*	CC BY-SA 4.0	*https://doi.org/10.17632/vsjrkrdgwj.1*
*Chassis*	*.stl*	CC BY-SA 4.0	*https://doi.org/10.17632/vsjrkrdgwj.1*
*Leg_3mm*	*.stl*	CC BY-SA 4.0	*https://doi.org/10.17632/vsjrkrdgwj.1*
*Leg_20mm*	*.stl*	CC BY-SA 4.0	*https://doi.org/10.17632/vsjrkrdgwj.1*
Testbed	.f3d	CC BY-SA 4.0	*https://doi.org/10.17632/vsjrkrdgwj.1*
*Build instruction*	*.jpg*	CC BY-SA 4.0	*https://doi.org/10.17632/vsjrkrdgwj.1*

(1) *Top* – part ensuring proper alignment between the light source and optical sensor (in-line), also responsible for flattening and removal of air gaps between both. The top mount is designed to directly hold collimation-ready LEDs (*i.e.*, from Thorlabs' mounted LED series) without requiring a separate lens tube clamp, such as the SM1TC. The top part is a flat mounting plate with a central circular hole for inserting a realistic Thorlabs’ mounted LED shape. The hole in the top of the enclosure has the same diameter as an SM1TC tube, allowing users to insert an LED effortlessly. In our experimental case, it accommodates a mounted NIR LED M810L3 (*Thorlabs*, USA).

(2) *Chassis* – mainframe of the testbed. This is where the tissue sample, measured 110 x 110 mm (maximum size), is placed during the experiment. The RX (*i.e.*, optical sensor or photodetector module) is placed at the bottom, directly attached to the tissue sample.

(3) Four detachable legs that lift the chassis and top parts, like LEGO pegs. As mentioned previously, this leg enables using different RX sizes without reprinting the entire testbed. The legs are installed at the four corners of the chassis, elevating it above the RX. They serve both as support and as a buffer, allowing the RX to fit underneath. In our case, we provide two types of legs:

• *Leg_3mm* – first option of the chassis support, allowing attachment of the desired sensor.
• *Leg_20mm* – second option of the chassis support, allowing attachment of the desired sensor.

(4) Testbed – complete design of the 3D-printable testbed, importable, and created using Autodesk Fusion360 (Fig. 3b).

## 4 Bill of materials summary

All components shown are fully 3D-printable using Fused Deposition Modeling (FDM) or Fused Filament Fabrication (FFF). The vast majority of commercially available 3D printers can fabricate all parts in a single print session. Furthermore, no additional purchased parts are required. Resin printing is also a viable option. However, parts might need to be printed separately.

Table 5 summarizes the amount of materials required by each part. For this study, all items were printed using PLA filament (Premium PLA, *Raise3D*, China). Material costs were calculated based on retail prices of $36.99 per kilogram. In our case, all components used to construct this 3D-printable testbed were sourced from materials already available in our laboratory.

**Table 5:** Bill of materials summary.

**Designator**	**Component**	**Number**	**Cost per unit −currency**	**Total cost −** **currency**	**Source of materials**	**Material type**
*Top*	PLA	1	$36.99 per kg	$1.10	https://www.3dprima.com	biodegradable thermoplastic
*Chassis*	PLA	1	$36.99 per kg	$4.50		
*Leg_3mm*	PLA	4	$36.99 per kg	$0.40		
*Leg_20mm*	PLA	4	$36.99 per kg	$0.60		

It should be noted that the reported total hardware cost of approximately $6.6, presented in Table 5, corresponds solely to PLA material consumption for in-house 3D printing. No additional fees (*e.g.,* service or machining) were incurred, as all components were printed using laboratory-owned printers. The proposed test-bed is entirely 3D-printable and does not require aluminum mechanics or other commercial optomechanical hardware. Consequently, the material cost of PLA represents the relevant and complete fabrication cost of the test-bed itself.

In summary, unlike optical benches, the proposed testbed is envisioned as an alternative approach for niche tests, offering a lightweight, compact, user-friendly solution and a low-cost fabrication method costing less than $100. This solution clearly addresses *RQ2* as outlined above.

Although 3D printing can be carried out in-house (if it is available), the university’s centralized 3D printing service from the fabrication laboratory (FABLAB) is also a viable option. For instance, we have FABLAB in our university (https://www.oulu.fi/en/university/fab-lab-oulu) providing services for equipment, including laser cutters, 3D printers, 3D scanners, vinyl cutters, a CNC milling machine, and electronic fabrication equipment. However, they might charge users based on local pricing policies (*e.g.*, filament weight and type); therefore, the reported total cost in Table 5 will not be relevant, but it is still expected to remain well below $100.

## 5 Build instructions

**Fig. 4:**
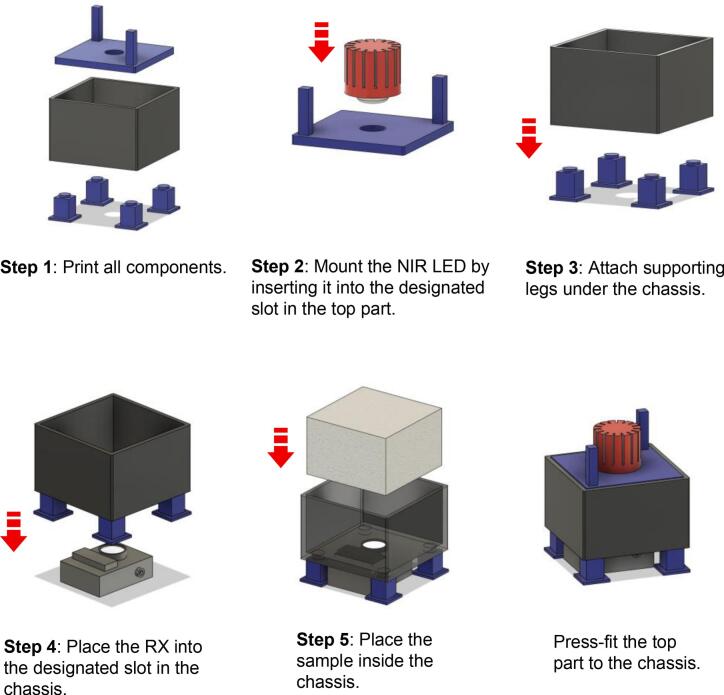
Visual depiction of a step-by-step build instruction, available in the repository.

This section outlines the step-by-step process for assembling the 3D-printable testbed. The design emphasizes accessibility and is easily assembled using consumer-grade 3D printers. A visual guide showing the step-by-step build instructions from 1 to 5 is provided in the repository (Fig. 4).

Meanwhile, the detailed steps are elaborated as follows:

***Step 1: Print all components***.

**Fig. 5:**
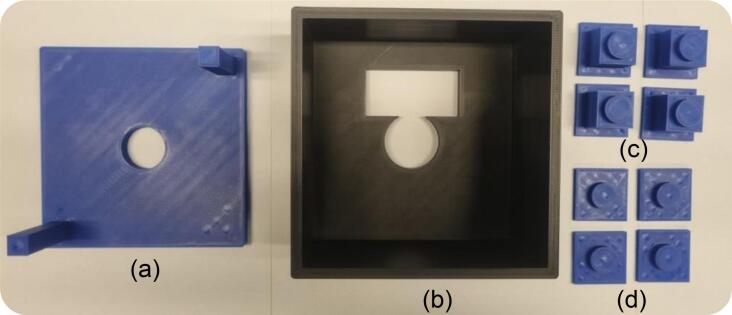
A photograph showing all components is printed: (a) *Top*; (b) *Chassis*; (c) *Leg_3mm*; (d) *Leg_20mm*.

• Download the STL files provided in the repository (see the Design Files Summary section): *Top, Chassis, Leg_3mm, and Leg_20mm*.
• Print them using PLA filament, which is recommended for its ease of printing and durability, with an Infill of approximately 20–30 % (Fig. 5).

***Step 2: Mount the LED (TX) into the top part***.

**Fig. 6:**
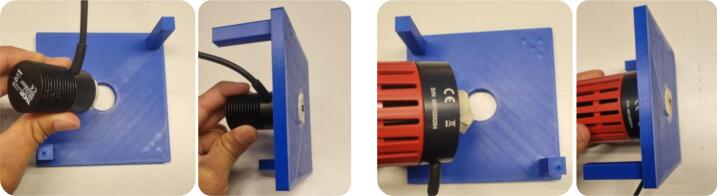
A photograph showing the LED inserted into the circular hole in the top part.

• The top part is a flat mounting plate with a central circular hole. It features two poles that function as hand grips, allowing the user to press the LED into the sample and release easily. Afterward, insert the Thorlabs’ mounted NIR LED into the circular hole in the top part (Fig. 6). The hole is dimensioned to match the outer diameter of the SM1 lens tube.

***Step 3: Attach the four detachable legs to the chassis***.

**Fig. 7:**
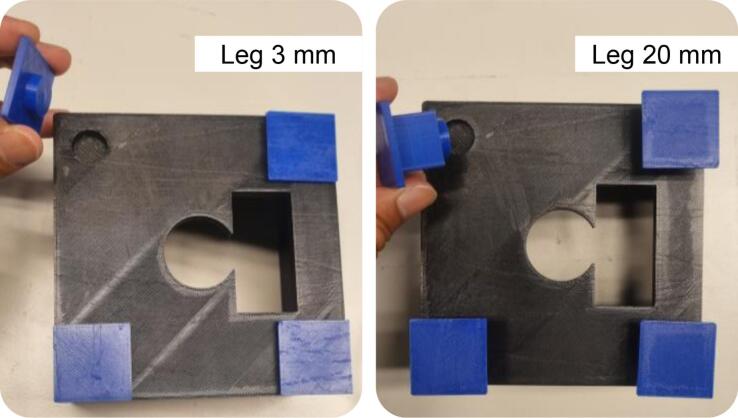
A photograph showing the legs is attached in the designated slot. The 3D-printable testbed accommodates both RX types by using interchangeable leg heights. The bottom part of the chassis features four corner holes for attaching the support legs via a LEGO-style press-fit mechanism, ensuring a secure fit. This modular construction enables users to quickly switch between different leg heights (*e.g.*, 3 mm or 20 mm) based on the type of RX used, without reprinting the entire enclosure.
• The leg height should be selected based on the type of RX used: if using the S121C (with a compact form factor), install the 3  mm legs (*leg_3mm*). Meanwhile, if using the PDA36A-EC (with a larger body), install the 20 mm legs (*leg_20mm*). These modular legs elevate the chassis, keep it considerably stable and flat on the experimental surface, and maintain consistent alignment along the optical axis. Therefore, make sure all four legs are securely attached to prevent tilting or shifting during the experiment.

• The main body of the 3D-printable testbed is a cubic-form chassis designed to house the sample during experiments. In addition to holding the sample, the chassis serves a dual function as a light-shielding enclosure, minimizing interference from ambient light sources. The chassis base also features four holes for modular legs (Fig. 7).

***Step 4: Install the RX into the chassis***.

**Fig. 8:**
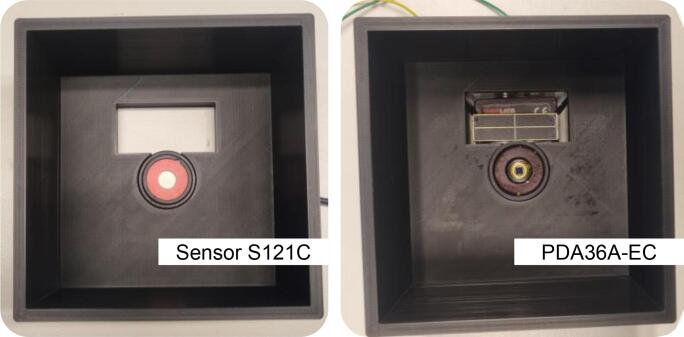
A photograph showing the RX is installed at the bottom of the chassis.

• As previously described, the RX can be either an optical sensor (*i.e.*, S121C) or a photodetector (*i.e.*, PDA36A-EC), depending on the specific type of measurement being conducted. The S121C is recommended for measuring absolute optical power (in mW or dBm) or power density (in mW/cm^2^). In contrast, the PDA36A-EC, a photodiode with built-in amplification, is more suitable for detecting modulated data, making it ideal for OWC-focused experiments.
• Afterward, secure the RX into the designated bottom slot of the 3D-printable testbed (Fig. 8). Manual alignment with the optical axis is not necessary, as the RX slot is pre-aligned with the NIR LED, which is mounted in the top part, ensuring precise vertical alignment by design. In addition to the RX sensor slot, the base includes a rectangular cut-out adjacent to the PDA36A-EC position, specifically designed to accommodate a photovoltaic (PV) cell. This configuration enables users to evaluate joint data and power transmission across biological tissue or phantoms, similar to what was previously demonstrated in our previous work [27]. The slot is designed for the dedicated RX, as mentioned above. Therefore, modifications may be needed for a larger RX.

***Step 5: Insert the Samples into the chassis***.

**Fig. 9:**
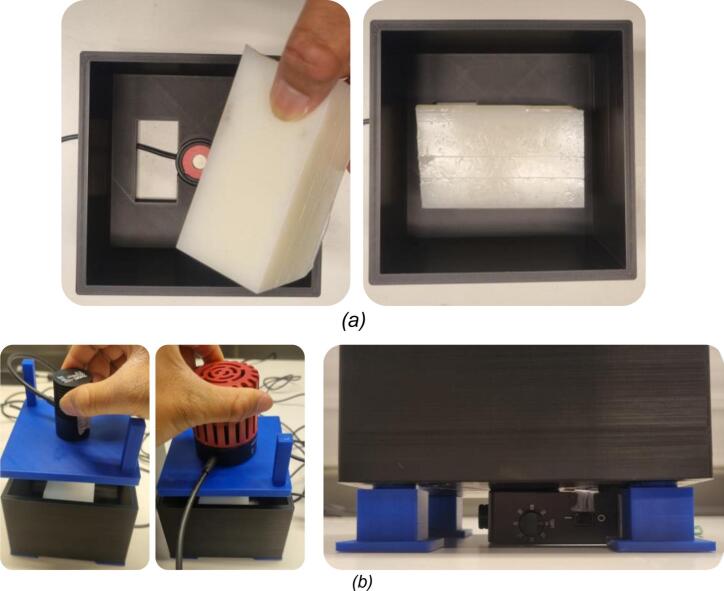
A photograph showing: (a) the sample is placed in the chassis; (b) the last step of testbed preparation.
• Carefully lower the top part with the mounted LED onto the chassis and press down gently, ensuring it sits evenly (Fig. 9b). The two side poles on the top part serve as handles, allowing controlled pressing and releasing and ensuring consistent contact with the sample.

• As mentioned earlier, it is highly recommended to adjust the sample (phantom or biological tissue) size to a maximum of 110  mm × 110  mm to ensure it fits properly within the chassis slot. Additionally, the phantom’s cross-sectional area should be significantly larger than the active sensing area of the photodetector used; for instance, the S121C or the PDA36A-EC has a circular area of 9.5 mm. Ensuring the sample fully covers the sensor helps avoid edge effects.
• Insert the sample into the designated slot (Fig. 9a).

***Step 6: Conducting Measurements***.

Once all components are installed, you are ready to perform measurements. Before starting, ensure the following electrical connections are correctly established:

• Connect the mounted LED to an LED driver circuit. In this study, we employed the LED driver module DC2200 (*Thorlabs*, USA).
• If using the S121C, connect its output to an optical power meter to measure received optical power. In this study, we used PM100D (*Thorlabs*, USA). Meanwhile, if using the PDA36A-EC, connect it to an oscilloscope or digital signal processing unit for analyzing modulated signals or waveform reception.
• To validate the light-shielding effectiveness of the enclosure, perform a baseline ambient light test: measure the received signal or optical power across the sample tissue with ambient room lighting ON, and repeat the measurement with ambient lighting OFF. Compare the results to confirm that the enclosure blocks external light, enabling accurate experimentation even under typical room lighting conditions.
• It should be noted that when working with NIR LEDs, users should be aware that the emitted light of the NIR LED is often invisible to the human eye, making it difficult to detect exposure. In our previous setup, using a horizontal testbed mounted on an optical bench, the sample was not enclosed or sealed, making it more vulnerable to ambient light interference. On the other hand, since NIR light is invisible to the naked eye, we sometimes use a smartphone camera to confirm whether the LED is emitting. Even though the 3D-printable testbed provides ambient shielding, it is still strongly recommended to wear certified laser safety glasses specifically designed for the wavelength in use. For example, Thorlabs offers certified protective eyewear for various NIR bands (the catalog can be found here: *https://www.thorlabs.com/newgrouppage9.cfm?objectgroup_id=762*). Always verify the optical density rating and wavelength range of the eyewear before use. These NIR LED sources can be set to operate at low power. Still, safety glasses should be worn during experiments.

## 6 Operation instructions

This section provides step-by-step guidance on using the 3D-printable testbed.

***Step 1: Pre-Operation Checklist***.

• Ensure all 3D-printed parts are assembled.
• The TX, RX, and four legs are properly placed in the designated slots.
• When using *ex-vivo* porcine tissue, ensure that the sample is fresh, properly hydrated, and correctly positioned within the chassis. If an optical phantom is used instead, care must be taken to ensure its surface is clean and free of dust, as dust can scatter or absorb light, affecting measurement accuracy. Maintaining proper sample condition and placement is essential for obtaining reliable and reproducible experimental results.
• Confirm that all wires are correctly connected to the LED driver, optical power meter, oscilloscope, or data acquisition system.

***Step 2: Powering On and Running Experiments***.

• Double-check the LED driver settings to ensure they match the LED specifications.
• Double-check the optical power settings; the step-by-step can be found here [22] before beginning data collection.
• As previously elaborated, NIR LEDs can emit invisible radiation. It is recommended to use appropriate NIR safety glasses during experiments.
• Press “ON” the LED driver to activate the LED.
• Conduct measurements according to your experimental protocol to achieve the experimental goals.
• Periodically check that the 3D-printable testbed has not shifted during the experiment.

***Step 3: Post-Experiment Procedures***.

• After completing the measurements, all supplies to the measurement devices and the LED driver should be safely powered down.
• Follow laboratory safety protocols when handling samples. The *ex-vivo* porcine sample should be replaced as it is a single-use sample. Meanwhile, the optical phantom should be removed and cleaned, as it is reusable.
• After the samples are removed, the chassis can be wiped clean with a soft cloth. When using gel or liquid optical phantoms or using *ex-vivo* porcine samples, ensure that all residues within the chassis are thoroughly cleaned to prevent contamination or damage. The RX and TX should be cleaned properly and inspected before the next use.

## 7 Validation and characterization

***Aims and Goals 1***.

**Fig. 10:**
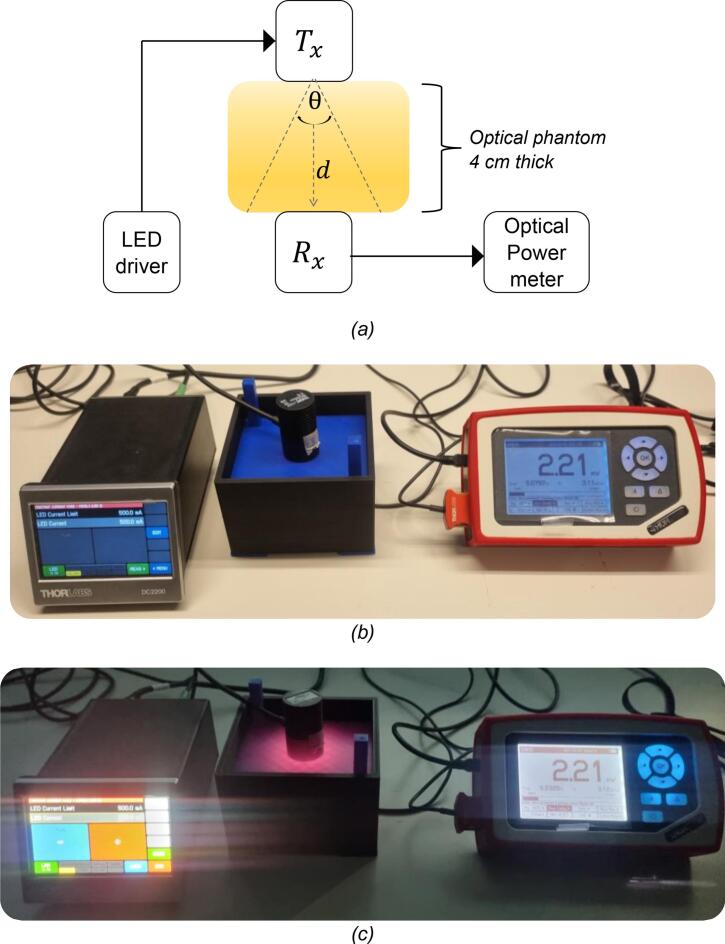
Experimental setup: (a) diagram block; (b) measurements under the laboratory room lighting is “ON”; and (c) the laboratory room lighting is “OFF.”.

To address *RQ1*, we characterize and validate the 3D-printable testbed by demonstrating its ability to effectively block ambient light. This ensures that in-body OWC experiments can be performed safely and repeatably under standard room-lighting conditions without compromising measurement accuracy. We performed optical power measurements using an optical sensor connected to the optical power meter console. The recorded parameters are absolute power (in mW) and power density (in mW/cm^2^). In this paper, we do not characterize the PDA36A-EC data-reception metrics, such as bit error rate (BER), as our focus is solely on optical power measurements. This choice was based on the understanding that ambient light variations can affect the received optical signal and, thus, indirectly affect BER performance. Therefore, the optical power serves as a reliable metric for evaluating the performance of the 3D-printable testbed under varying ambient lighting conditions. Given that OWC across biological tissue is highly sensitive to even small variations or fluctuations in light intensity (as observed in the misalignment experiment [19]), the S121C provides the accuracy required for baseline validation of the testbed. Fig. 10(a) shows the experimental setup used for the experiment, conducted under two lighting conditions: with the laboratory lamp turned ON (Fig. 10b) and OFF (Fig. 10c).

We used two types of NIR LED as TX, *i.e.*, 810 nm and 850 nm (Thorlabs M810L3 and M850LP1, respectively). The transmitted power of the NIR LEDs was varied using a programmable LED controller module (*i.e.*, LED Driver DC2200) from 100 % to 80 %, 60 %, 40 %, and 20 % of the LED capacity. For instance, the M810L3 mounted LED has a maximum optical output of 375 mW at 100 % capacity (500 mA forward current). To achieve lower output levels, we adjusted the current proportionally via the LED driver. For example, operating the LED at 80 % capacity (400 mA) yields an approximate transmitted power of 300 mW. Meanwhile, the M850LP1 NIR LED has a maximum current rating of 1500  mA. Varying the operating current to 80 %, 60 %, 40 %, and 20 % of its full capacity corresponds to 1200 mA, 900 mA, 600 mA, and 300 mA, respectively.

The optical sensor and NIR LED were placed in the bottom slots of the chassis and on the top plate. In both scenarios, the TX was aligned with the RX through the sample placed inside the chassis of the 3D-printable testbed. In this test, we used an optical phantom previously applied in previous studies [27], [28], [29]. The phantom was sized to fit within the chassis of the 3D-printable testbed, covering the sensor aperture fully for accurate optical power detection. It mimics the average optical properties of realistic human soft tissue, which is fabricated from polyvinyl chloride plastisol (PVCP), forming a solid medium. It exhibits very stable chemical properties, enabling long-lasting use. The phantom was developed based on the work of [30], with optical parameters spanning the 400–1100  nm range [31]. The measured attenuation and propagation behavior observed in the optical phantom are expected to reasonably reflect *in-vivo* conditions, as both are governed by fundamental light–tissue interactions such as photon absorption, scattering, and diffusion through tissues. We assume that experimental results from phantom-based measurements can also serve as a direct baseline for future *in-vivo* experiments, where the same physical mechanisms will apply.

The PM100D recorded the optical power received by the S121C. For the measurement, the PM100D was configured to the correct wavelength corresponding to the NIR LED used in the experiment, ensuring accurate calibration at 850 nm or 810 nm. Other important settings are listed in Table 6.

**Table 6:** The configuration of the optical power meter PM100D.

**Settings**	**Values**
*Ring (Rng)*	Auto
*Wavelength (λ)*	850 nm / 810 nm
*Attenuation (Att.)*	0.00 dB
*Bandwidth (BW)*	HI
*Aperture (ϕ)*	9,500 nm
*Line filter*	50 Hz

Each condition was repeated five times for consistency. Before each measurement, the PM100D was zeroed with the NIR LED turned “OFF”. The standard deviation (*Std Dev*) for each set of repeated measurements was calculated to assess measurement results consistency, as described in (1).

(1) $$\sigma =\sqrt{\frac{1}{N-1}\sum_{i=1}^{N}{\left(x_{i}-\overset{¯}{x}\right)}^{2}}$$

Where,

σ is the *Std Dev*.

$x_{i}$ is the individual measurement.

$\overset{¯}{x}$ is the mean of the measurements.

N is the number of measurements.

When the lamp was turned OFF, the measured optical power served as the baseline, representing the ideal condition with minimal ambient light interference. When the lamp was turned on, the testbed’s enclosure effectively shielded the phantom and receiver from external light sources. By establishing a controlled baseline with and without ambient light, we infer that the BER would degrade under such conditions as well.

In the analysis, we highlight the measured absolute power (in mW). The measurement results are shown in Table 7, Table 8 for 810 nm and 850 nm NIR LEDs, respectively. For instance, in Table 7, the measured optical power using 100 mA LED driving current reference through a 4  cm-thick optical phantom under laboratory lamp ON conditions across five repeated tests was 0.436 mW, 0.436 mW, 0.435 mW, 0.439 mW, and 0.440 mW, respectively, yielding a Mean and *Std Dev* of 0.437 mW and 2.17 µW, respectively. Meanwhile, under laboratory lamp OFF conditions, the values were 0.44 mW, 0.439 mW, 0.439 mW, 0.439 mW, and 0.442 mW, respectively. The corresponding Mean and *Std Dev* are 0.44 mW and 1.30 µW, respectively. The percentage of *Std Dev* relative to the mean for 100 mA is 0.3 % and 0.5 % for lamp OFF and lamp ON conditions, respectively. A low *Std Dev* indicates that measurement readings are highly consistent, with minimal fluctuation between each measurement. In practical terms, variations of less than 1 % are typically considered acceptable. Across all current levels (*i.e.*, 100–400 mA), the *Std Dev* remained low, at less than 0.65 % of the Mean, demonstrating strong measurement consistency. Additionally, the average optical power difference between the lamp ON and OFF states was minimal (≤1%), indicating that ambient light does not significantly interfere.

**Table 7:** Measurement of received optical power (in mW) across a 4 cm-thick optical phantom under 810 nm NIR light.

**LED Current Setting**	**Laboratory Room Condition**	**Mean (mW)**	**Standard Deviation (mW)**	**Percentage**
400 mA	Lamp OFF	1.802	0.004472	0.25 %
	Lamp ON	1.794	0.011402	0.64 %
300 mA	Lamp OFF	1.366	0.005477	0.40 %
	Lamp ON	1.358	0.008367	0.62 %
200 mA	Lamp OFF	0.911	0.002345	0.26 %
	Lamp ON	0.9054	0.00498	0.55 %
100 mA	Lamp OFF	0.4398	0.001304	0.30 %
	Lamp ON	0.4372	0.002168	0.50 %

**Table 8:** Measurement of received optical power (in mW) across a 4 cm-thick optical phantom under 850 nm NIR light.

**LED Current Setting**	**Laboratory Room Condition**	**Mean (mW)**	**Standard Deviation (mW)**	**Percentage**
1200 mA	Lamp OFF	10.522	0.026833	0.26 %
	Lamp ON	10.612	0.043243	0.41 %
900 mA	Lamp OFF	8.274	0.013416	0.16 %
	Lamp ON	8.322	0.019235	0.23 %
600 mA	Lamp OFF	5.712	0.004472	0.08 %
	Lamp ON	5.732	0.004472	0.08 %
300 mA	Lamp OFF	2.904	0.005477	0.19 %
	Lamp ON	2.912	0.004472	0.15 %

As shown in Table 8, across all current levels of NIR LED at 850 nm (*i.e.*, 300–1200 mA), the variation in measured optical power is minimal, with *Std Dev* remaining below 1 % of the Mean in all cases. For instance, at 1200  mA, the average received power was 10.612 mW (Lamp ON) and 10.522 mW (Lamp OFF). The Lamp ON condition yielded slightly higher variation (0.41 %) than Lamp OFF (0.26 %); both *Std Dev* remained low. Interestingly, at lower currents, such as 300 mA and 600 mA, the variation is even smaller. For instance, at 600  mA, the deviation under lamp OFF was 0.08 %. Overall, the findings are in line with those at 810 nm, particularly regarding the consistency of the received optical power under both lamp ON and OFF conditions (Table 8). Moreover, the measurement results confirm the testbed’s capability to effectively isolate optical path interference under normal laboratory lighting. The raw data of the measurement campaigns are provided in Supplementary File **1.**

**Fig. 11:**
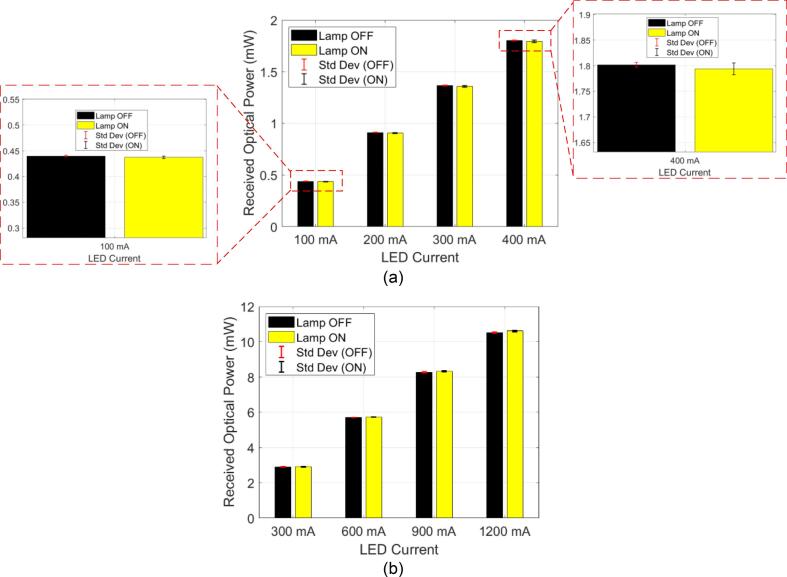
Measurement results: (a) Data observed at NIR light 810  nm, with the zoomed-in section highlighting the *Std Dev* at the 100  mA and 400 mA settings; (b) Corresponding measurements at 850  nm NIR light under identical conditions.

Fig. 11(a) and 11(b) depict the average received optical power at 810  nm and 850  nm, respectively, under both lamp ON and lamp OFF conditions across varying drive currents. The graph showed no significant increase in baseline when ambient lighting was present, confirming that the testbed is highly suitable for repeatable in-body OWC experimentation without the ergonomic drawbacks of working in fully dark conditions. In the initial version of the 3D-printable testbed, the top plate was printed in blue (as shown in Fig. 10a). To optimally improve ambient light shielding (toward the noise floor), it is recommended to print the top plate in black, matching the chassis color, as darker colors provide better suppression of ambient light (see our additional experimental setup in the Appendix, Fig. 18).

### 7.1 Aims and Goals 2

**Fig. 12:**
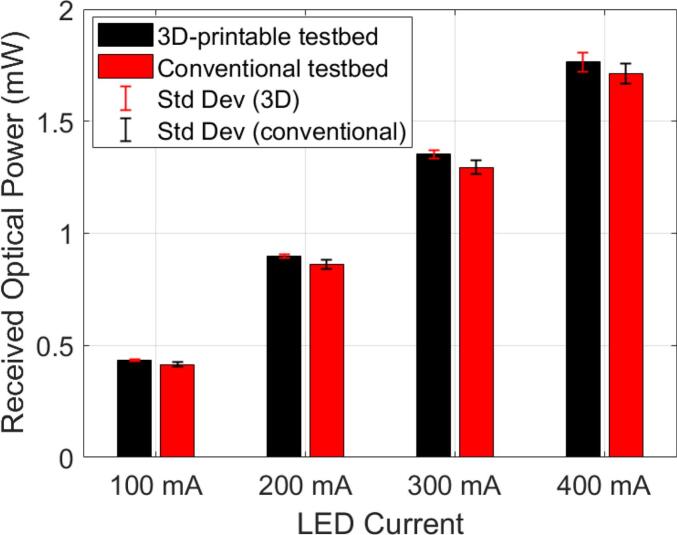
Comparison between the 3D-printed testbed and the traditional optical bench over five repeated assembly cycles conducted at ambient lighting-free*.*

To address *RQ3*, we evaluate the plug-and-play repeatability of the designed 3D-printable testbed by comparing the consistency results with those of a standard horizontal optical bench (conventional testbed). In both setups, an NIR LED acted as the TX, placed opposite a RX behind a 4 cm-thick optical phantom as previously used. For each configuration, the TX and RX were completely disconnected and reassembled five times to simulate routine laboratory activities, including plugging, unplugging, and repositioning. After each reassembly, we measured the received optical power under the same conditions. The collected data were then compared to assess how well each platform maintains the mounted NIR LED – optical phantom – optical sensor geometry and to determine whether the 3D-printable testbed provides alignment consistency comparable to that of the traditional optical bench. Both tests were conducted in a dark room to ensure fair conditions. Fig. 12 shows the experimental results, indicating low *Std Dev*. The testbed design has been proven to support consistent TX–RX alignment and reproducible measurements across multiple sessions, even under repeated unplugging, re-plugging, and reassembly of the setup.

### 7.2 Hardware Limitations

**Fig. 13:**
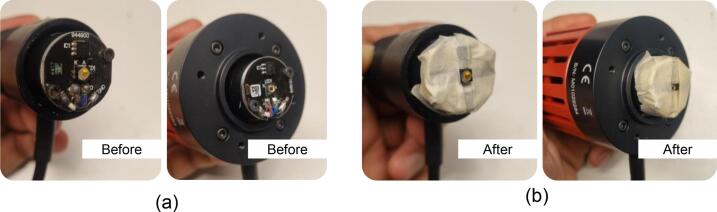
(a) Mounted LED’s surface; (b) Protective elements of a mounted NIR LED using adhesive tape. The insulating tape is applied to shield exposed electrical contacts from the moisture present in samples. The LED’s active optical surface is intentionally left uncovered. The tape-based approach offers both simplicity and a low-cost solution.
• **Effective Only Under Typical Laboratory Room Lighting**: We assume that standard optical laboratories are usually direct sunlight-free to ensure measurement reliability. The room is strictly protected from any ambient light noise. Therefore, the most relevant problem in our use case is ambient light from laboratory lighting. In our case, the detected ambient light levels were approximately 90 µW and 100 µW at 850 nm and 810 nm, respectively (see Appendix, Fig. 19). Although this ambient light from typical laboratory lighting conditions represents a relatively low-level signal, it may still introduce interference or baseline offsets in sensitive optical measurements involving the in-body OWC system if not adequately suppressed. The filaments and wall thicknesses in the current testbed design were intentionally selected to provide sufficient shielding under these typical indoor lighting conditions. Importantly, our measurements have already demonstrated that the enclosure with ∼ 30 % infill effectively suppresses ambient light. Our testbed is characterized by highly directional, with the detector oriented perpendicularly to the NIR source. The detector, on the other hand, is centrally positioned and relatively far from the case body. The air gap at the top is minimal, so incident light from the ceiling cannot reach the tissue sample directly. All of which further reduces the impact of random ambient light that might penetrate the enclosure sides. However, the situation may differ in laboratories with high illumination from laboratory lighting or significant incident sunlight (ambient optical power > 100 µW), and researchers may use rooms with direct or diffuse sunlight during optical measurements. Sunlight may provide stronger ambient illumination than standard laboratory lighting. In such conditions, intense light may pass through a 3D-printed chassis, especially if it is printed with low infill or higher-transmittance materials. As a practical solution, we consider that a full mechanical redesign of the testbed is unnecessary. Instead, improved ambient light shielding may be achieved by adjusting the printing parameters (*e.g.*, using 100 % infill). Additional mitigation to achieve adequate shielding could be achieved by manufacturing the chassis from a lower-transmittance filament (*e.g.*, black PETG, ABS, or carbon-filled PLA) and/or by increasing the wall thickness. Details on possible materials for such purposes were discussed in [32]. Our 3D-printable testbed has not yet been evaluated under strong sunlight, as this was not feasible at the time of the study due to seasonal conditions. The experiments took place in winter, characterized by frequent snowfall, foggy skies, and weak sunlight intensity, which is generally lower than standard laboratory lighting. Still, conducting systematic evaluations under strong sunlight would be more beneficial for understanding the effectiveness of ambient light shielding. These assessments are ideally scheduled for the summer, when strong sunlight is mostly available.
• **No temperature meter slot**: Tissue safety is a critical consideration when exposing biological samples to NIR light. As specified by safety standards for active implantable medical devices (ISO 14708–1:2014), the temperature increase at the tissue–device interface is generally limited to approximately 2 °C above normal body temperature to ensure safe long-term use [33], [34]. Studies on IED emphasize that potential tissue heating from wireless links (whether using optical or radio) should be explicitly managed in experimental setups. Therefore, in the context of in-body OWC experiments, tissue heating should ideally be monitored and kept within the same safety margin as defined in the standard. However, the 3D-printable testbed features a fully enclosed design with no additional openings, maximizing shielding of indoor ambient light. A dedicated slot or port for temperature probes is not yet available. As a result, the testbed does not support in situ temperature measurement during NIR exposure in its present version. A new version of the 3D-printable testbed might include a dedicated clip-on probe channel to allow compliant temperature monitoring without compromising light isolation. This feature would make it easier to demonstrate compliance with the tissue-heating threshold and better align the experimental platform (*i.e.*, proposed 3D-printable testbed) with ISO safety assessment practices.
• **Mechanical stability**: The existing 3D-printable testbed has lower mechanical inertia than a typical optical setup bench. The lightweight design enhances portability but may be more prone to minor shifts when touched accidentally or when subjected to lateral force from cables, potentially compromising mechanical stability during extended measurements. We addressed this by securing the chassis to the work surface with double-sided adhesive tape (Tesa Double Tape 1.15 mm, *Tesa SE*, Germany), preventing displacement during the measurement sessions (Fig. 14). The optical sensor was also secured using the same method. While this method is effective, it might not be suitable for professional deployment. The solution may include a more secure stabilization option, such as a rubber anti-slip pad attached to the bottom of the support legs, the use of a mounting clamp, or an optional bolt-down base plate, as illustrated in Fig. 15(a), (b), and (c), respectively.

While the presented testbed offers unique advantages in terms of open source, cost, portability, ease of use due to alignment consistency, protects the ambient light, and has potential applications for the educational sector, our current design also has several limitations:

• **Limited LED compatibility**: The current design is tailored for Thorlabs’ mounted NIR LEDs, such as the M810L3 or similar models from the collimation-ready LED series, available at *https://www.thorlabs.com/newgrouppage9.cfm?objectgroup_id=2692*. Specifically, the top plate in our design is dimensioned to match the mechanical envelope of this LED family. As a result, the current configuration does not natively accommodate other TX devices (*e.g.*, LEDs from different manufacturers, laser diode modules, or fiber-optic collimators). Supporting such a TX would require modifying the LED-housing region of the top plate to ensure proper mechanical fit and optical alignment. Surprisingly, this design modification is straightforward and does not affect the rest of the testbed structure, since the top plate’s overall footprint remains fixed; only the diameter of the TX hole needs to be adjusted. Future updates to the repository might include interchangeable top-plate options that support TX sources beyond Thorlabs’ mounted NIR LEDs.
• **Restricted Misalignment Testing**: The in-body communication system may consider misalignment experiments to represent real-world situations, for instance, when medical staff fail to align the TX with the RX during operations (improper alignment setting), or when there is a slight movement of in-body devices due to an accidental case, *e.g.*, the in-body device is tilted or shifted [19]. Our design accounts for the built-in mechanism that prioritizes vertical precision for strictly ideal transmission experiments, *i.e.*, the alignment setting. This testbed focuses on establishing a measurement baseline to align the TX and RX. Considering misalignment settings, a novel approach is required to create an alternative version of the 3D-printable testbed.
• **Fixed detector slot size**: The bottom portion of the chassis is designed to fit a specific range of RX sizes. Thicker, larger, or non-standard optical sensor or photodetector packages may not be compatible without redesigning the enclosure base and leg part. Without modification, it may limit flexibility for researchers using custom RX or employing RX from different vendors.
• **No heater capability**: In previous testbed designs [22], it was possible to integrate heating elements, such as a chamber with heat blower properties, to maintain the *ex-vivo* porcine sample at approximately 37 °C, closely mimicking human physiological temperature. The current testbed is fabricated using PLA filament, which begins to soften around 60–65 °C. However, the compact size and material limitations of the current 3D-printed testbed present challenges for active temperature control. It does not include a built-in heating system. Therefore, the heating procedure must be handled externally if needed, and with caution regarding material tolerances.
• **Lack of Electrical Circuit Shielding**: The surface of Thorlabs’ mounted LEDs contains exposed electrical contacts (*i.e.*, wiring and electronic component pads), as shown in Fig. 13(a), which may be vulnerable to direct contact with moist biological samples or phantoms. To prevent accidental electrical short circuits, the mounted NIR LED must be properly insulated, while leaving the LED’s active area surface uncovered to ensure unobstructed optical transmission. We chose a very thin adhesive tape (301E, *3M^TM^*, USA) as a temporary insulator, as shown in Fig. 13(b), for the following practical considerations. First, it does not introduce a considerable optical path length. Its thickness is on the order of tens to a few hundred micrometers, which we consider negligible; thus, it neither alters the LED–tissue distance nor measurably affects the received optical power. Even with careful design, using a dedicated 3D-printed shield to protect the rear pads of the mounted NIR LED would inevitably increase thickness and further alter the optical spacing. Specifically, the adhesive tape is an industry-standard tape with good conformability to irregular surfaces and is well-suited for holding and sealing applications, making it an appropriate choice for covering the uneven surface of the rear pad of Thorlabs’ mounted NIR LED and providing considerable protection. Its shelf life of approximately 18 months is adequate for medium-term use. Second, there might be variability among mounted LEDs. Although Thorlabs’ mounted LEDs share a similar outer housing, the emitter size may differ between models. In our case, we used a mounted NIR LED with a 1 mm × 1 mm emitter size. Designing a universal 3D-printed shield that consistently fits all variants is challenging, as it would add more setup variations and become costly. Using a thin insulating layer could avoid this model-specific variability while keeping the top part of the 3D-printable testbed compatible with the entire LED family. Importantly, the top circular opening of the top part was precisely sized to match the Thorlabs’ mounted LED housing, ensuring consistent alignment and easy insertion/removal regardless of the temporary insulation method. We acknowledge, however, that this method may appear less professional than dedicated 3D-printed accessories. We may provide an optional 3D-printed insulating cap for users who prefer a permanent solution tailored to a specific LED model.

**Fig. 14:**
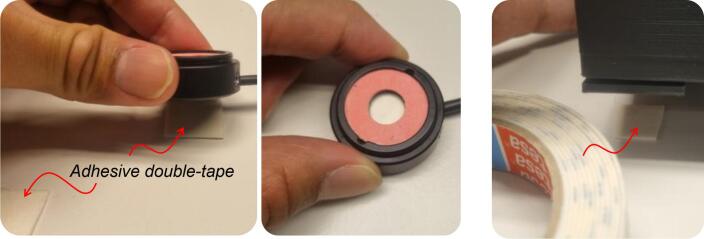
Current solutions for a 3D-printable testbed to address the issues related to mechanical stability.

**Fig. 15:**
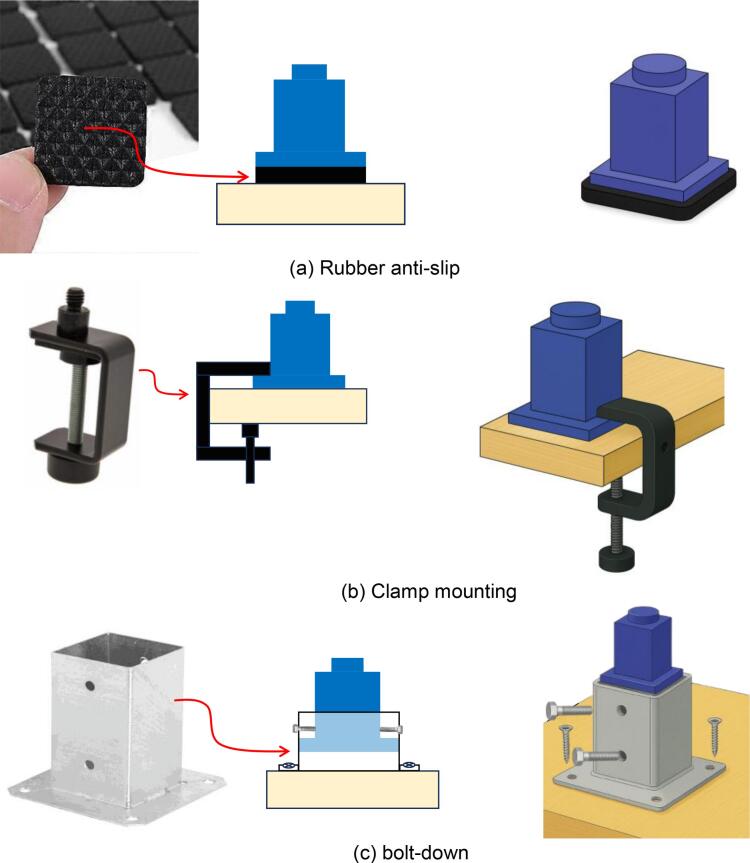
Possible solutions for enhancing mechanical stability: (a) Rubber anti-slip feet, which help absorb minor vibrations and prevent unintended minor movement caused by accidental contact. It can be directly attached to the bottom of the support legs; (b) Mounting clamp, which locks the support leg, allowing the testbed to be temporarily secured to a tabletop. This solution is better than rubber anti-slip, as it provides a more flexible stabilization option for uneven surfaces while remaining portable and easy to assemble. Meanwhile, (c) for semi-permanent installations, a bolt-down base plate can be an excellent option, enabling the testbed to be rigidly fixed with standard screws.
• **Long-term durability and fit tolerance:** Another limitation of the current 3D-printable testbed is the long-term mechanical wear caused by extensive repeated use. Over multiple assembly cycles, the press-fit interfaces may gradually loosen, especially the circular hole in the top plate that secures the NIR LED and the snap-fit leg sockets at the chassis base, which are designed like LEGO pieces. In our experiments, minor loosening of the LED interface was temporarily mitigated by applying a thin adhesive tape (301E, *3M^TM^*, USA) around the mounted LED body to restore a snug fit and suppress mechanical play (Fig. 16). While this works well during measurements, it is not suitable for long-term or professional deployment. Future testbed versions should feature a more innovative design to address the long-term durability and fit tolerance.

**Fig. 16:**
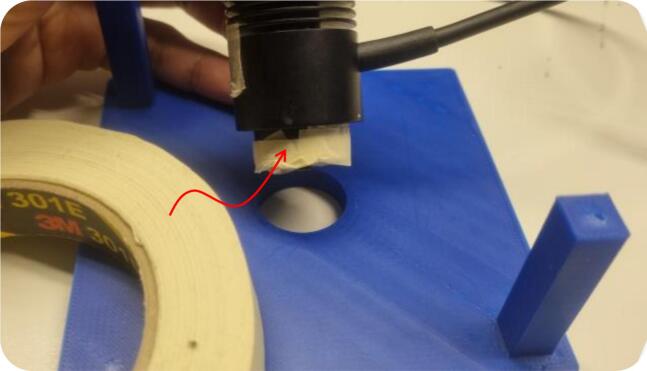
Current solutions for a 3D-printable testbed to address the issues related to a loose testbed interface.

### 7.3 Conclusion and Future Work

There has been growing interest in optical wireless communication (OWC) for in-body electronic devices (IEDs) in recent years, driven by its advantages over several limitations of traditional radio-frequency (RF) approaches, such as low electromagnetic interference (EMI) and operation in license-free spectral bands. The optical link in in-body OWC typically requires strict alignment due to its sensitivity to misalignment. Still, it provides additional benefits, such as secure, safe, and private medical data transmission. Typically, experimental testbeds using these conventional testbeds have two technical challenges. First, manually aligning the optical TX and RX is typically time-consuming and prone to error, leading to inconsistent results and potentially reducing data reproducibility. Second, it required conducting tests in low-light conditions to avoid interference from indoor ambient light. Both issues posed not only practical but also eye-safety challenges, particularly during extended experiment sessions. Prolonged work in dark environments can cause discomfort and eye strain, which may gradually reduce a researcher’s productivity.

This paper presents a cost-effective 3D-printable testbed for in-body OWC communications research. The modular design ensures precise vertical alignment of optical components and improves the efficiency of the experimental setup. By effectively shielding the testbed from ambient light, experimental reliability is increased while also eliminating the need for dark environments. This significantly improves ergonomic comfort, reducing eye strain and hence enabling longer, more comfortable research sessions. The customizable, open-source design makes it a practical and accessible solution across various research settings. The consistency observed across repeated plug-and-play cycles indicates that the 3D-printed testbed also delivers a reproducible experimental testbed comparable in stability to conventional optical benches. This 3D-printable testbed is therefore well-suited for routine laboratory measurements and educational activities, especially in environments where optical setups are frequently reconfigured.

However, despite the chassis design providing sufficient shielding for practical in-lab use, the fixed vertical alignment between the TX and RX does not accommodate experiments involving angular or lateral misalignment. Such requirements may be essential for advanced optical modeling or robustness evaluations against pointing errors, which could be a focus of future work. Furthermore, adapting the enclosure to accommodate other LED packages beyond Throlabs’ mounted LED may need mechanical modifications to the top housing.

## Ethics statements

The authors declare that this study did not involve any human subjects or animal experiments; therefore, no ethical approval was required. The authors developed the conceptual designs shown in Fig. 1, Fig. 15 (3D view). AI tools (ChatGPT/DALL·E, *OpenAI*, USA) were used solely to generate base graphical assets, which the authors then extensively modified.

## CRediT authorship contribution statement

**Syifaul Fuada:** Writing – original draft, Visualization, Validation, Investigation, Data curation. **Lukasz Surazynski:** Writing – review & editing, Visualization, Methodology, Formal analysis, Conceptualization. **Mariella Särestöniemi:** Writing – review & editing, Supervision, Resources, Conceptualization. **Teemu Myllylä:** Supervision, Resources, Project administration, Conceptualization. **Marcos Katz:** Writing – review & editing, Supervision, Resources, Funding acquisition.

## Declaration of competing interest

The authors declare that they have no known competing financial interests or personal relationships that could have appeared to influence the work reported in this paper.

## Figures and Tables

**Fig. 17:**
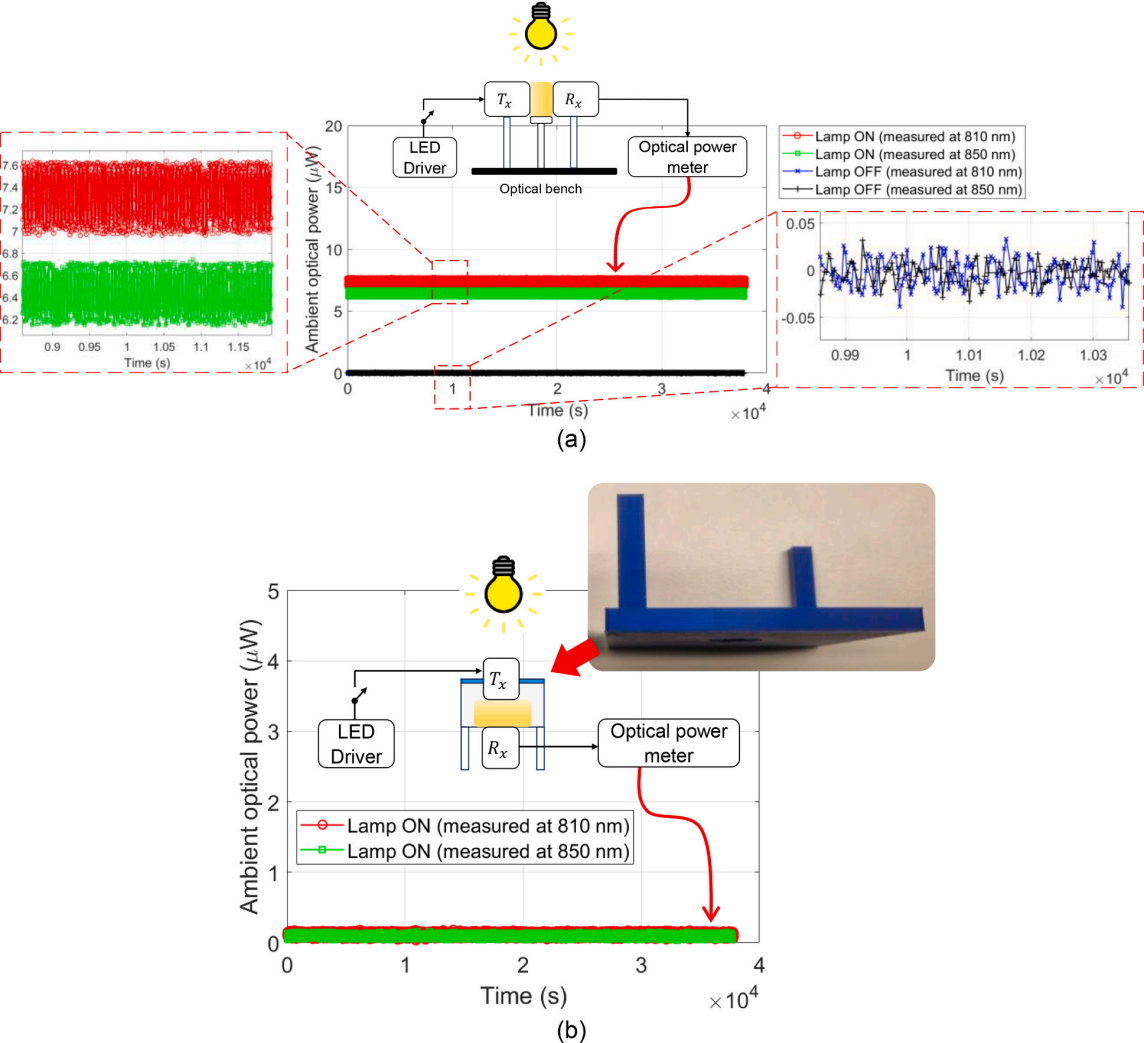
(a) Ambient light penetrating optical phantom measured in a prior testbed. A small background signal was detected when using the 810 nm and 850 nm reference wavelengths on the optical power meter, indicating that ambient indoor laboratory lighting can partially penetrate the optical phantom under the open measurement setup; (b) Ambient light measurement using the proposed 3D-printable testbed.

**Fig. 18:**
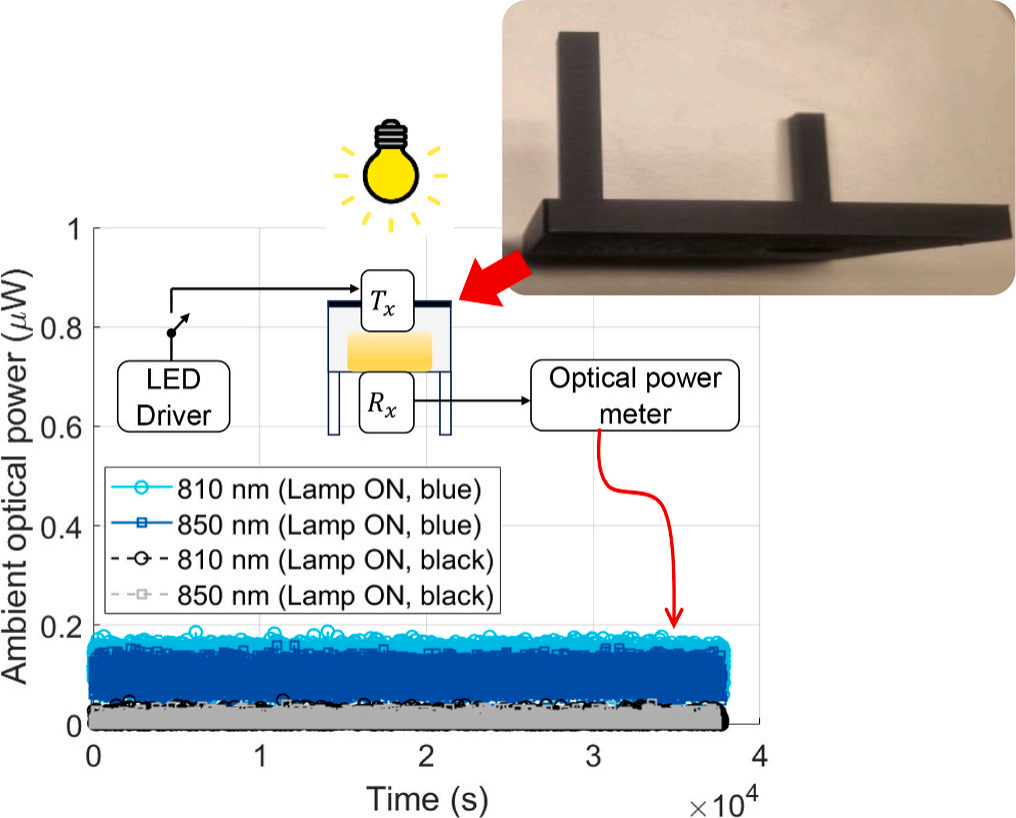
Performance comparison between blue- and black-colored 3D-printed top plates in suppressing ambient laboratory light. It shows that the use of a black top plate effectively replicates dark-laboratory conditions, as it suppresses ambient light to a level comparable to that in experiments conducted in a fully darkened environment.

**Fig. 19:**
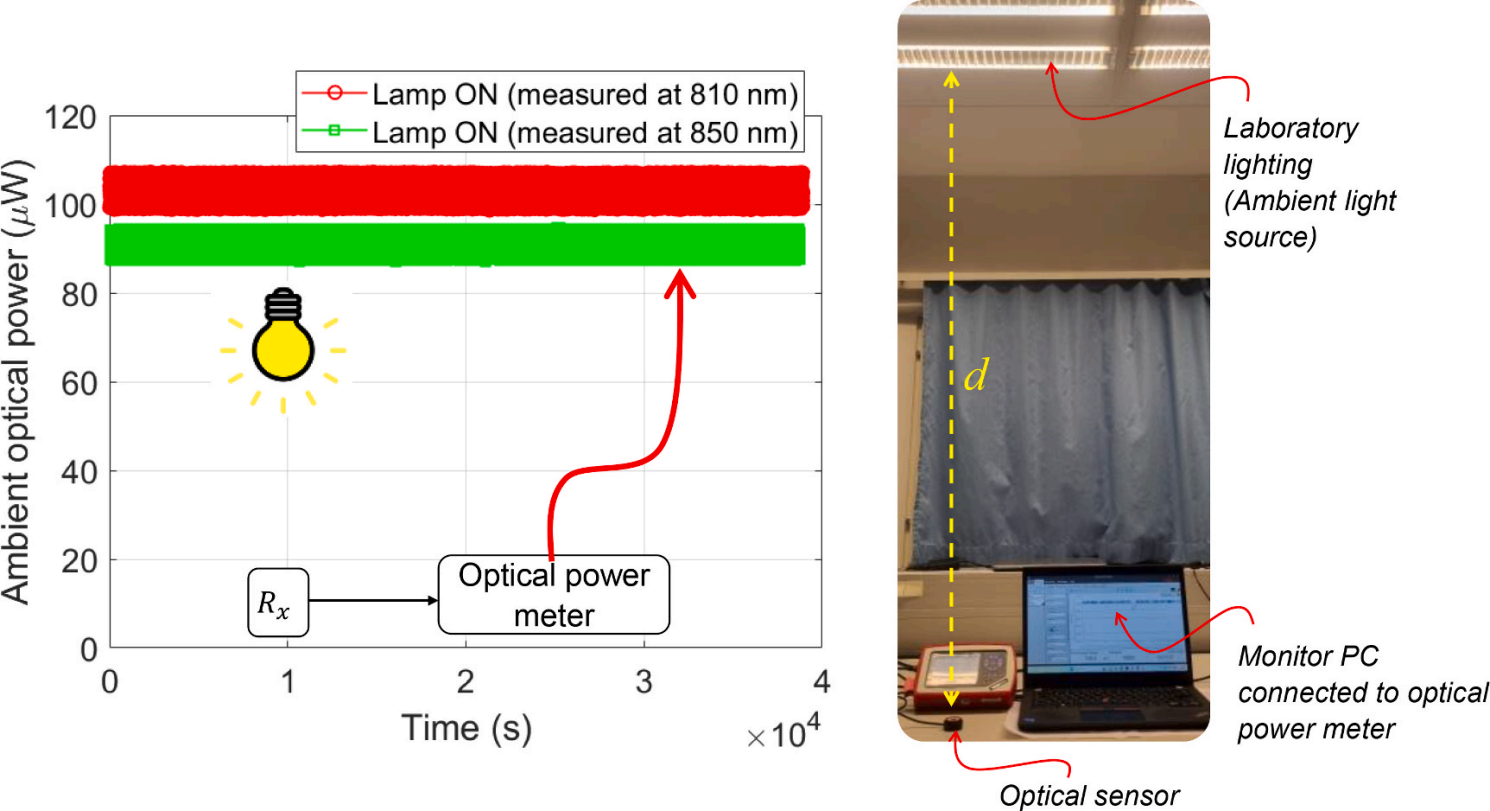
Ambient light measurements, obtained using an optical sensor connected to an optical power meter at 810 nm and 850 nm.

**Fig. 20:**
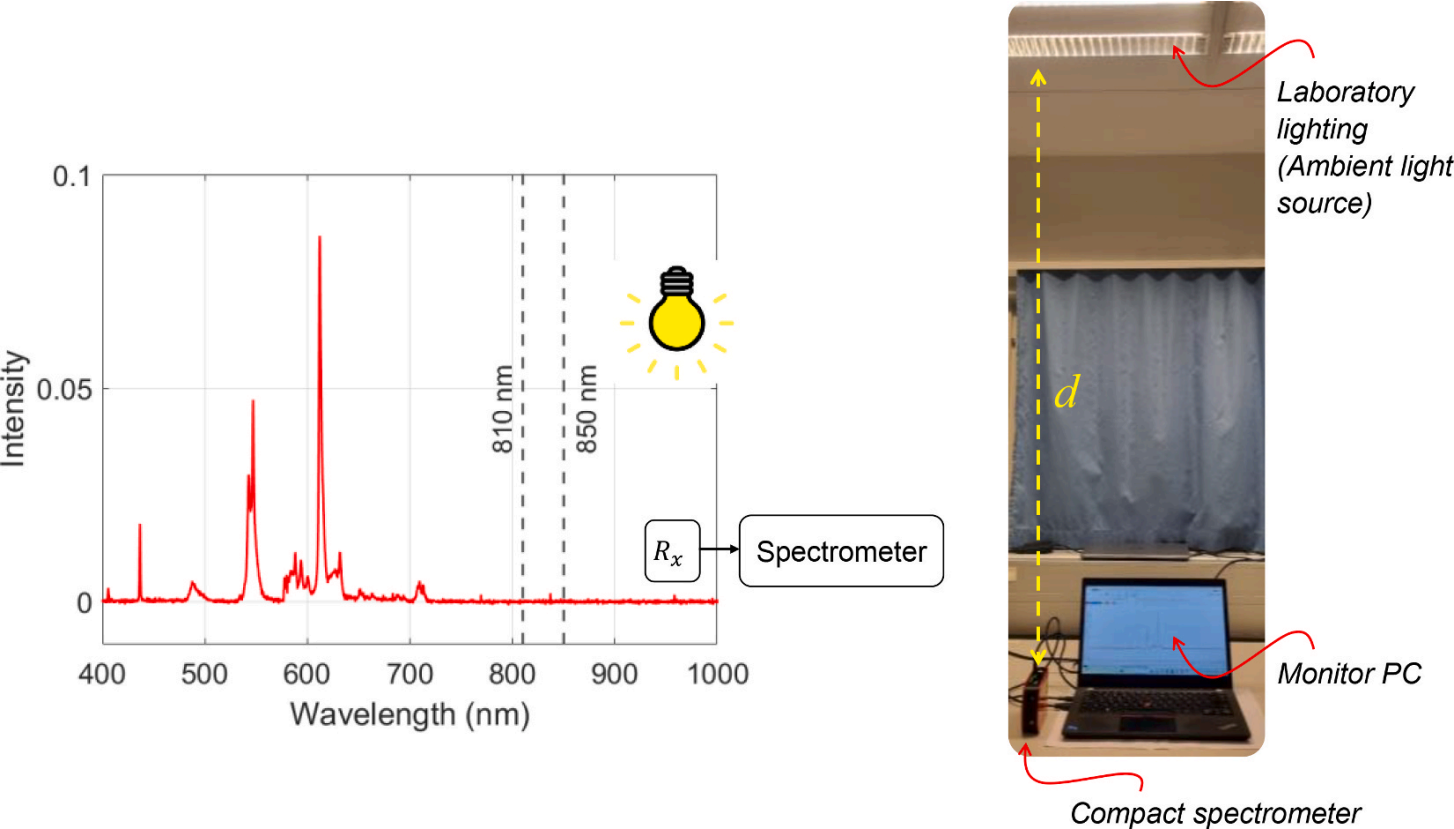
Spectral distribution of ambient laboratory lighting measured using a spectrometer with cosine correction over the 400–1000 nm wavelength range. The spectrum is dominated by visible-light emission, with only weak NIR components corresponding to the testbed's operating wavelengths, *i.e.*, 810 nm and 850 nm. The ambient light spectrum was measured using dedicated software (ThorSpectra v1.3.0/1.0.0, *Thorlabs,* USA) with the spectrometer operating in fixed-averaging mode (1000 averages). The integration time was set to 45,000 ms, and amplitude correction was disabled to detect low-level spectral components. To maintain consistency with the optical phantom’s spectral range, the ambient light spectrum was shown over 400–1000 nm, although data was recorded from 200–1000 nm.

## Appendix

This section discusses how ambient light from our laboratory lighting can affect the received-power measurements on our previous testbed. We measured the ambient optical power passing through the optical phantom using an optical sensor with an optical power meter. The NIR LED was completely switched off, and the optical phantom was kept in the same position as during the regular experiments. The optical power meter (configured separately for 810 nm and 850 nm) was connected to a computer running the corresponding control software (Optical power monitor version 5.0.4590.776, *Thorlabs*, USA), which logged optical power over time (10,000 data points) and exported the measurements as CSV files. In MATLAB, the time stamps and power readings were extracted. A graph of time (in seconds) versus ambient light power (µW) was plotted to visualize the appearance of the ambient signal received by the optical phantom under typical room-light conditions. Fig. 17(a) indicates that laboratory illumination affects the measurement, evidenced by a non-zero ambient optical power when the laboratory lights are switched on. This underscores the need for dedicated shielding in in-body OWC experiments. We then repeated the measurement on the 3D-printable testbed under the same lighting conditions. As shown in Fig. 17(b), the 3D-printable testbed nearly eliminates ambient light, *i.e.*, stays near zero, which is close to the instrument’s noise floor, thereby enabling accurate in-body OWC measurements even under typical laboratory lighting.

The laboratory lighting emission was also measured using the same equipment: an S121 connected to a PM100D optical power meter, configured for 810 nm and 850 nm wavelength settings. Measurements were performed at a distance equal to the separation between the ambient light source and the test bed. The measurement results, shown in Fig. 19, indicate that ambient light levels are approximately 90– 100 µW for these wavelength references. The spectral content of the ambient laboratory lighting in our case was characterized using a compact spectrometer (CCS200/M, *Thorlabs,* USA) equipped with a cosine corrector (CCSB1, *Thorlabs,* USA). Measurements were performed at the same distance between the laboratory light source and the work desk as used in the experiments. As shown in Fig. 20, the spectrum is dominated by strong emission peaks in the visible region, a typical characteristic of indoor lighting sources. In the NIR region, including wavelengths around 810 nm and 850 nm, only very weak spectral components are observed, consistent with the low ambient optical power levels measured using the S121C, as shown in Fig. 20. Although these NIR components are small, they may still contribute to a measurable baseline in sensitive optical experiments. Analysis of the spectral profile, as shown in Fig. 20, indicates that the ambient light source is likely fluorescent, showing distinct emission peaks and weak NIR signals, as illustrated based on https://www.evergreen-life.co.uk/health-wellbeing-library/natural-light-health-benefits/. Other common lighting options, such as halogen, incandescent, daylight, warm-white, or cool-white lamps, generally have broader spectra and tend to produce a stronger NIR background, which may affect measurements. However, the 3D-printable testbed, particularly when equipped with a black top plate, may still effectively shield against these lighting variations owing to its fully enclosed design and due to the selected color of the top plate, which is black and has a light-absorbing profile.

Time-series optical power recorded under identical room-lighting conditions with the NIR LED transmitter switched OFF. The measured signal remains at the instrument's noise floor, demonstrating the effective shielding against ambient light.
